# Who Are the Gypsies?

**Published:** 1883-03

**Authors:** 


### WHO ARE THE GYPSIES?

Charles G. Leland, who, as is well known, has made special studies of the Gypsies and their language, oontributes an illustrated paper to the April CENNURY on "Visiting the Gypsies," from which we quote the following:

In conclusion, I may briefly answer a question which many persons have put, "Who and what are the Gypsies?" To this, I reply that they are of a mixed Aryan and non-Aryan stock from Northern India, where they have been known since prehistoric times. In their own language they call themselves Rom, meaning husband; but the word may also have some affinity with *ramna*, meaning to roam or wander. I believe that I have been the first to prove that there is at the present day in India, among the one hundred and fifty kinds of wandering castes of that country, which are all Gypsies, one in particular which is there regarded as specially Gypsy, and which calls itself *Rom*, and which uses words not collected in any other Indian dialect. but which are used by the Gypsies of Syria, Turkey and Europe. This tribe is allied to, and is most probably only a more widely wandering branch of the Dom, who are also known as outcasts and Gypsies. When I speak of so many kinds of wanderers as Gypsies, and yet not identical with our own, I may make my meaning clearer by saying, that as all the tramps, peddlers, etc., who roam in our roads are still not Romany, so of all the Indian nomads. there is but one which in every particular, especially that of language, exactly corresponds to those whom I have described.

---

SIMPLE CURE FOR COLD FEET.—The following remedy for cold feet is recommended by the *Fireman's Journal* for sedentary sufferers, as well as policemen, car drivers and others who are exposed to the cold: All that is necessary is to stand erect and very gradually to lift one's self up upon the tips of the toes, so as to put all the tendons of the foot at full strain. This is not to hop or jump up and down, but simply to rise—the slower the better—upon tiptoe, and to remain standing on the point of the toes as long as possible, then gradually coming to the natural position. Repeat this several times, and by the amount of work the tips of the toes are made to do in sustaining the body's weight, a sufficient and lively circulation is set up. A heavy pair of woolen stockings drawn over thin cotton ones is also a recommendation for keeping the feet warm. and at the same time preventing their becoming tender and sore.

